# Technical and clinical considerations of a physical liver phantom for CT radiomics analysis

**DOI:** 10.1002/acm2.14309

**Published:** 2024-02-22

**Authors:** Bino Abel Varghese, Steven Yong Cen, Kristin Jensen, Joshua Levy, Hilde Kjernlie Andersen, Anselm Schulz, Xiaomeng Lei, Vinay Anant Duddalwar, David John Goodenough

**Affiliations:** ^1^ Department of Radiology Keck Medical Center University of Southern California Los Angeles California USA; ^2^ Department of Physics and Computational Radiology Oslo University Hospital Oslo Norway; ^3^ The Phantom Laboratory Greenwich New York USA; ^4^ Department of Radiology and Nuclear Medicine Oslo University Hospital Oslo Norway; ^5^ Department of Radiology George Washington University Washington, D.C. USA

**Keywords:** computed tomography, imaging, phantom study, radiomics, radiomics phantom

## Abstract

**Objective:**

This study identifies key characteristics to help build a physical liver computed tomography (CT) phantom for radiomics harmonization; particularly, the higher‐order texture metrics.

**Materials and methods:**

CT scans of a radiomics phantom comprising of 18 novel 3D printed inserts with varying size, shape, and material combinations were acquired on a 64‐slice CT scanner (Brilliance 64, Philips Healthcare). The images were acquired at 120 kV, 250 mAs, CTDIvol of 16.36 mGy, 2 mm slice thickness, and iterative noise‐reduction reconstruction (iDose, Philips Healthcare, Andover, MA). Radiomics analysis was performed using the Cancer Imaging Phenomics Toolkit (CaPTk), following automated segmentation of 3D regions of interest (ROI) of the 18 inserts. The findings were compared to three additional ROI obtained of an anthropomorphic liver phantom, a patient liver CT scan, and a water phantom, at comparable imaging settings. Percentage difference in radiomic metrics values between phantom and tissue was used to assess the biological equivalency and <10% was used to claim equivalent.

**Results:**

The HU for all 18 ROI from the phantom ranged from −30 to 120 which is within clinically observed HU range of the liver, showing that our phantom material (T3‐6B) is representative of biological CT tissue densities (liver) with >50% radiomic features having <10% difference from liver tissue. Based on the assessment of the Neighborhood Gray Tone Difference Matrix (NGTDM) metrics it is evident that the water phantom ROI show extreme values compared to the ROIs from the phantom. This result may further reinforce the difference between a structureless quantity such as water HU values and tissue HU values found in liver.

**Conclusion:**

The 3‐D printed patterns of the constructed radiomics phantom cover a wide span of liver tissue textures seen in CT images. Using our results, texture metrics can be selectively harmonized to establish clinically relevant and reliable radiomics panels.

## 1 INTRODUCTION

Radiomics, defined as the high throughput extraction of mineable quantitative metrics from routine clinical images, is emerging as a powerful tool to aid diagnostic, prognostic, treatment evaluation applications in a variety of diseases, particularly cancer. However, poor procedural standardization and generalization of results,^1^, ^2^, ^3^ that is, the observation of wider variations in radiomic studies using multicenter imaging data, hinder the clinical translation of radiomics.^4^, ^5^, ^6^, ^7^, ^8^, ^9^, ^10^, ^11^, ^12^ Choe et al. showed that the variation of image acquisition variables has a greater effect on the reproducibility of radiomic metrics than interobserver variability.^13^ Propagation of such inconsistencies through the radiomics workflow eventually affects the extracted radiomic metrics. This leads to a scenario where the differential performance may be mainly due to heterogeneity of the data acquired rather than the underlying pathophysiology.^1^, ^2^, ^5^, ^13^, ^14^

With a wide variety of imaging scanners that are available in different centers, it is not practical to have uniform protocols across different hospitals and scanner models. Phantom studies have been used to assess the reliability of radiomic metrics, with variations in the radiomic process.^6^, ^15^, ^16^, ^17^ Various phantoms have been established over time, each characterizing different properties such as size, shape, texture, etc. For example, initiatives by the Radiological Society of North America (RSNA) Quantitative Imaging Biomarker Alliance (QIBA)^18^ have resulted in the development of Chronic obstructive pulmonary disease (COPD)/Asthma phantom, Ultrasound Shear Wave Speed (US‐SWS)‐Digital Phantoms, Diffusion‐weighted Imaging (DWI) phantoms, etc. Additionally, in some cases, virtual phantoms, or digital reference objects (DROs), have been useful for evaluation of software packages that are used to derive quantitative imaging biomarkers such as the PET/CT DRO, FMRI DRO, QIBA DCE‐MRI DRO, etc. While DROs have been very useful in studying the effects of post‐processing filters on quantification, the application has been limited to select imaging and simpler metrics such as shape and size. Zhao et al., developed an anthropomorphic phantom^19^ of the thorax and simulated 3D tumors of different shapes by placing them in different position in the phantom to assess reliability. However, owing to the homogenous texture of the materials used, texture analysis could not be performed. Reliability assessment of texture phantoms were performed using a water phantom, and Gammex American College of Radiology (ACR) phantom with water inserts, by Lo et al.^20^ and Lu et al.,^21^ respectively. However, in these studies weak texture and low SNR prevented a reliable assessment. The Credence Cartridge Radiomics (CCR) Phantom^6^ is currently a widely used texture phantom. However, this phantom can only assess global textures and it is specific to the lung. In addition, the inter‐scanner variability depends on choice of cartridge material and the radiomic metric being assessed. Finally, the phantom's cuboidal shape is not ideal for CT imaging and prone to artefacts. More recently, National Institute of Standards and Technology (NIST) reported on a simple method for evaluating of changes in tumor volume and size over time^22^ using vials and diapers. However, once the objects are prepared, they cannot be used again, and the reproducibility is limited.

Currently, some radiomics studies report the use of phantoms for harmonization/ standardization prior to radiomics analysis.^6^, ^23^ However, the assessment uses different phantoms, different standardizations, different validation data (lung vs. other organs) and varying subset of features, that is, texture versus shape/size. Therefore, it is difficult to reliably draw conclusions from different phantom studies particularly when the data variability is large. For example, while using the CCR^6^ texture phantom, Larue et al., reported no significant effect of tube current on radiomics features,^24^ whereas Mackin et al., showed significant effects of tube current on homogeneous textures than tissue textures of the CCR phantom.^7^ This observation warrants the need for much more granular assessment of radiomic metrics simultaneously within a single phantom, single study, and their associations with imaging variables.

In this study, we expand on recently published work aimed at establishing minimum thresholds of image quality for conducting reliable radiomics analysis.^25^ Here, a novel CT imaging phantom built for radiomics reliability assessment, overcoming some of the limitations of prior phantoms, has been presented. In particular, a cylindrical radiomics phantom, in which variations in size, shape, and texture can be assessed simultaneously has been described. Textural differences using variations in patterns and materials have been explored. Assessment of the performance of developed phantom shows its suitability to capture higher‐order texture metrics and equivalence to their values measured on in‐vivo liver CT scans.

## 2 METHODS

### 2.1 Phantom design

**FIGURE 1:**
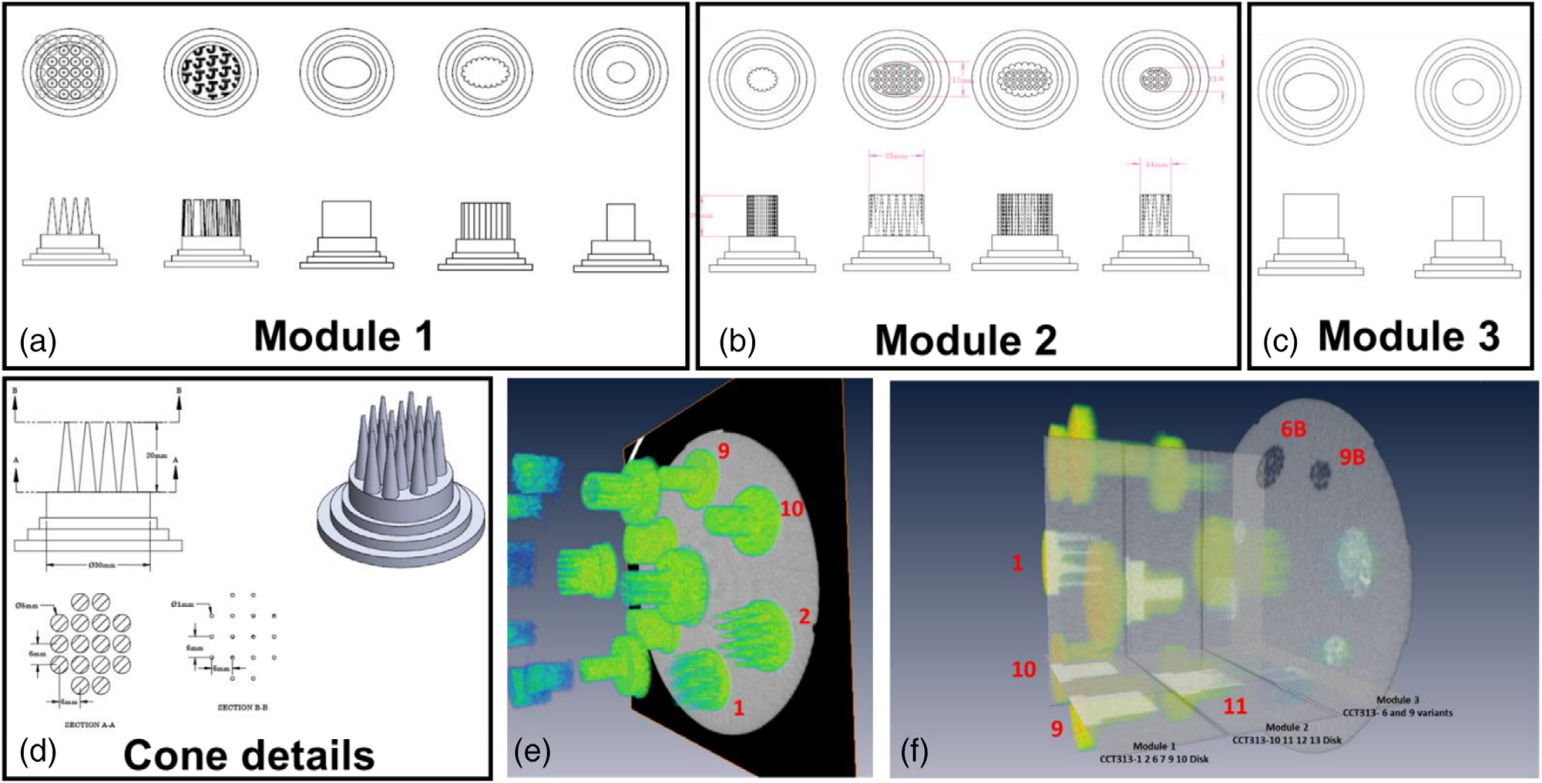
Details of the texture patterns created as part of the multimodule radiomics phantom. (a–c) shows the schematics of the features portrayed in Modules 1, 2, and 3, respectively. (e) shows the variations in the z‐direction created using cone structures (e) and (f) show volume rendering of the CT volumes of the radiomics phantom with multiple stacked modules.

**FIGURE 2:**
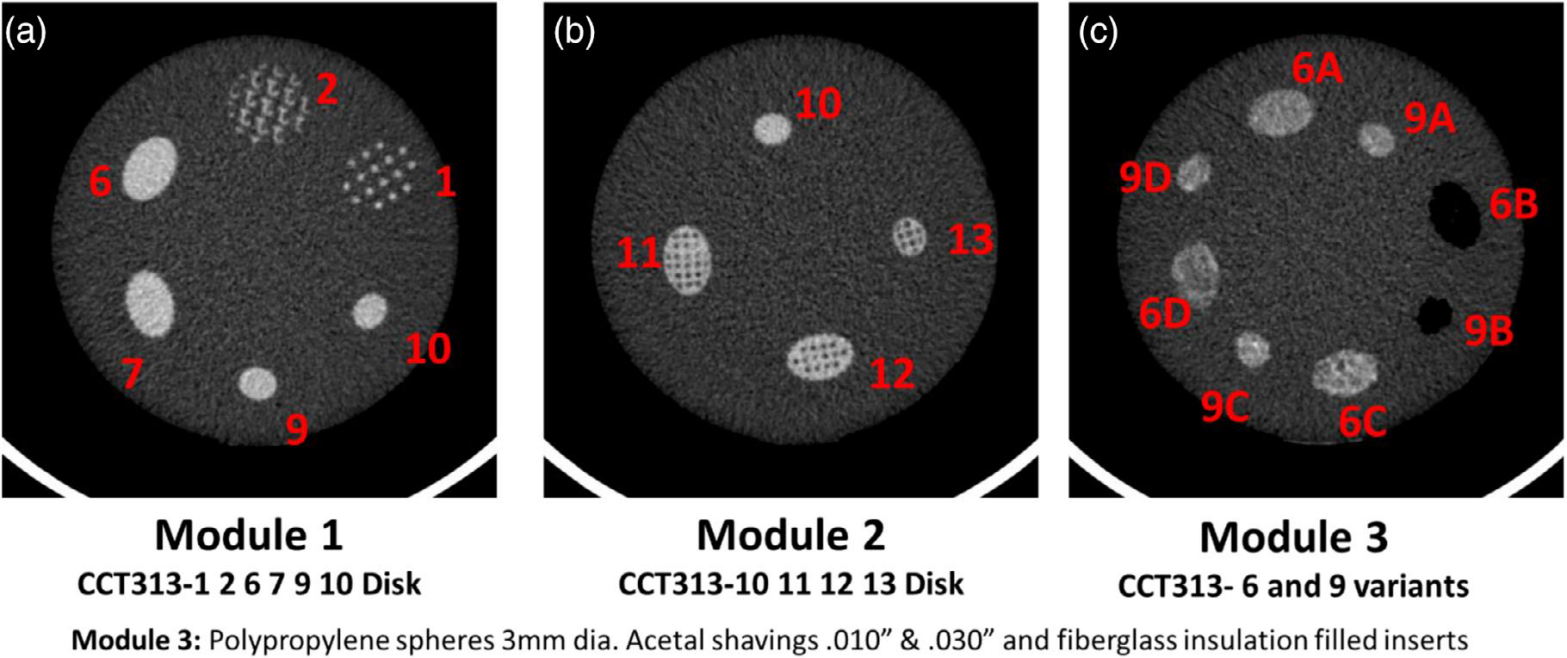
2D CT images of the central slice of the CT volume showing the details of the texture patterns created as part of the multimodule radiomics phantom. The radiomics phantom (The Phantom Laboratory, Salem, NY), consisted of three modules, namely, module CCT313 with patterned disks labelled by the manufacturer as 1 2 6 7 9 10 (Module 1), module CCT313 with patterned disks 10, 11, 12, 13 (Module 2) and module CCT313‐ with patterned disks that are variants of 6 and 9 (Module 3). Patterns 6A and 9A are made up of interspersed 0.010′ acetal shavings. Pattern 6B and 9B are made up of interspersed 3 mm diameter polypropylene spheres. Patterns 6C and 9C are made up of interspersed 0.030′ acetal shavings. Finally, Patterns 6D and 9D are made up of interspersed fiberglass insulation filled inserts.

The radiomics phantom (The Phantom Laboratory, Salem, NY), as shown in Figure 1, consisted of three modules, namely, module CCT313 with patterned disks labelled by the manufacturer as 1 2 6 7 9 10 (Module 1), module CCT313 with patterned disks 10, 11, 12, 13 (Module 2) and module CCT313‐ with patterned disks that are variants of 6 and 9 (Module 3) (Figure 1). Modules 1 and 2 comprised of disks with varying packing density, size, shape, and edges. Module 3 comprised of disks with the pattern similar to disk 6 and disk 9 from Module 1 but patterns 6A and 9A are made up of interspersed 0.010′ acetal shavings. Pattern 6B and 9B are made up of interspersed 3 mm diameter polypropylene spheres. Patterns 6C and 9C are made up of interspersed 0.030′ acetal shavings. Finally, Patterns 6D and 9D are made up of interspersed fiberglass insulation filled inserts. Yet another novelty of the design is the cone architecture of the features making up the disks. This design builds in a variability of the features in the z‐dimension (Figure 2) unseen in other phantoms.^6^ From a construction standpoint, the difference in the acrylic based 3D printed materials and the clear urethane surrounding the cones provided the contrast texture.

### 2.2 CT imaging and reconstruction

A 64‐slice CT scanner (Brilliance 64, Philips Healthcare, Andover, MA) was used for scanning. The images were acquired at 120 kV, 250mAs, CTDIvol of 16.36 mGy, 2 mm slice thickness and reconstructed using iDose1 (iDose with level 1, Philips Healthcare, Andover, MA). iDose is an iterative reconstruction algorithm developed by Philips Healthcare that provides low‐noise CT reconstructions compared with the standard, that is, the filtered backprojection (FBP) algorithm. The optimal treatment of noise by the iDose algorithm at low signal levels, reduce the noise propagated through reconstruction algorithms and improve image quality. In this study, level 1 of iDose was used. According to published literature, iDose1 reconstruction can achieve a noise reduction of 10.6% compared to an FBP reconstruction of the same CT acquisition. To benchmark the performance of the newly designed radiomics phantom, a commercial anthropomorphic liver phantom (The Phantom Laboratory, Salem, NY) and in‐vivo patient were scanned with the same settings. The water phantom is a PMMA cylinder, 32 cm in diameter, 12 cm in length and filled with water, provided by Canon for CT scanner calibration. The anthropomorphic liver phantom is a simplified representation of a human torso with a 25 cm × 35 cm oval shape and a length of 15 cm. The phantom body is cast from RANDO® tissue simulating material and contains simplified liver and bone structures. The liver is cast out of a higher density tissue material and the vertebrae and ribs are cast from simulated bone materials. The phantom has seven 5 cm diameter holes for holding insert rods. Three holes pass through the RANDO® material and four holes pass through the liver. A male patient (68 years old) with inoperable metastases from colorectal cancer receiving palliative treatment was scanned with the same protocol as the phantoms. Written informed consent was obtained.

### 2.3 Region of interest segmentation

**FIGURE 3:**
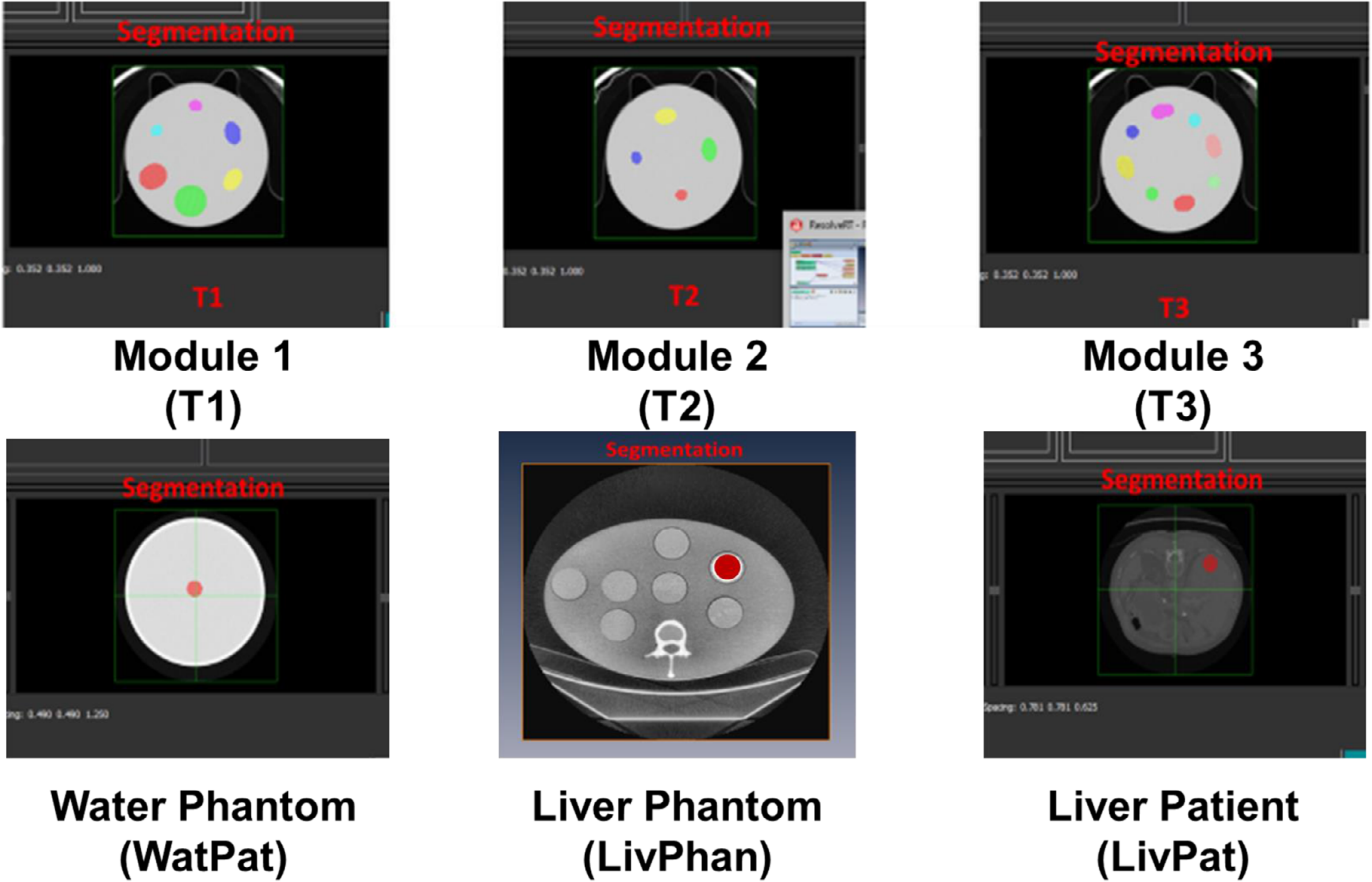
Manual segmentation and definition of ROI across the radiomics phantom, water phantom, liver phantom, and in‐vivo liver scan.

Manual delineation of the multiple 3D regions of interest (ROI) on the CT scans was performed using an open‐source software package CapTk (version‐1.8.1; https://www.med.upenn.edu/cbica/captk/). A total of 21 ROI were manually contoured by an experienced user (Figure 3). On the new test radiomics phantom, a centrally placed circular ROI ∼1 cm diameter was manually segmented over five adjacent CT slices of each of the 18 texture disks featured across the three modules. Specifically, six ROI capturing the six texture disks of module 1, four ROI capturing the four texture disks of module 2, and eight ROI capturing the eight texture disks of module 3 were segmented. The number of ROI on the new radiomics phantom totaled 18. On the water phantom, a single centrally placed circular ROI ∼1 cm diameter each was manually segmented over five adjacent CT slices. On the anthropomorphic liver phantom also a single circular ROI ∼1 cm diameter each was manually segmented over five adjacent CT slices encompassing one of the holes that pass through the liver. Similarly, in the in‐vivo liver CT scan also a single circular ROI ∼1 cm diameter each was manually segmented over five adjacent CT slices where purely liver tissue was present. Regions with obvious blood vessels were avoided.

### 2.4 Radiomics extraction

Radiomics features were computed from each ROI using CapTk 360 radiomic metrics featured within the CaPTk radiomics platform comprising of first‐order statistical metrics of texture such as intensity, histogram; first‐order statistical metrics of morphology such as shape, size; second‐order statistical metrics of texture such as grey level co‐occurrence matrix (GLCM), grey level size zone matrix (GLSZM), grey level run length matrix (GLRLM), neighboring grey tone difference matrix (NGTDM); and higher‐order statistical metrics of texture such as local binary patterns (LBP) were extracted from each ROI. To eliminate the variations from the HU range of different CT scanners, a fixed HU range [−1000, 1500] with a fixed number of grey‐level discretization bins, that is,10 was adopted. No other imaging preprocessing tasks were performed before feature extraction.

### 2.5 Statistical analysis

In this study, the HU mean, standard deviation as well as five NGTDM feature values derived from each of the 18 ROI were individually compared to the average value derived from the ROI in the patient liver scan. Bar charts and heat maps were used to illustrate the similarities. The percentage of difference between the value from phantom ROI and the value from the patient liver scan were also calculated and illustrated in a heatmap. SAS 9.4 (SAS Institute, Cary, North Carolina) was used for all statistical analyses.

## 3 RESULTS

### 3.1 Tissue density equivalence of the radiomics phantom to in‐vivo liver tissue

**FIGURE 4:**
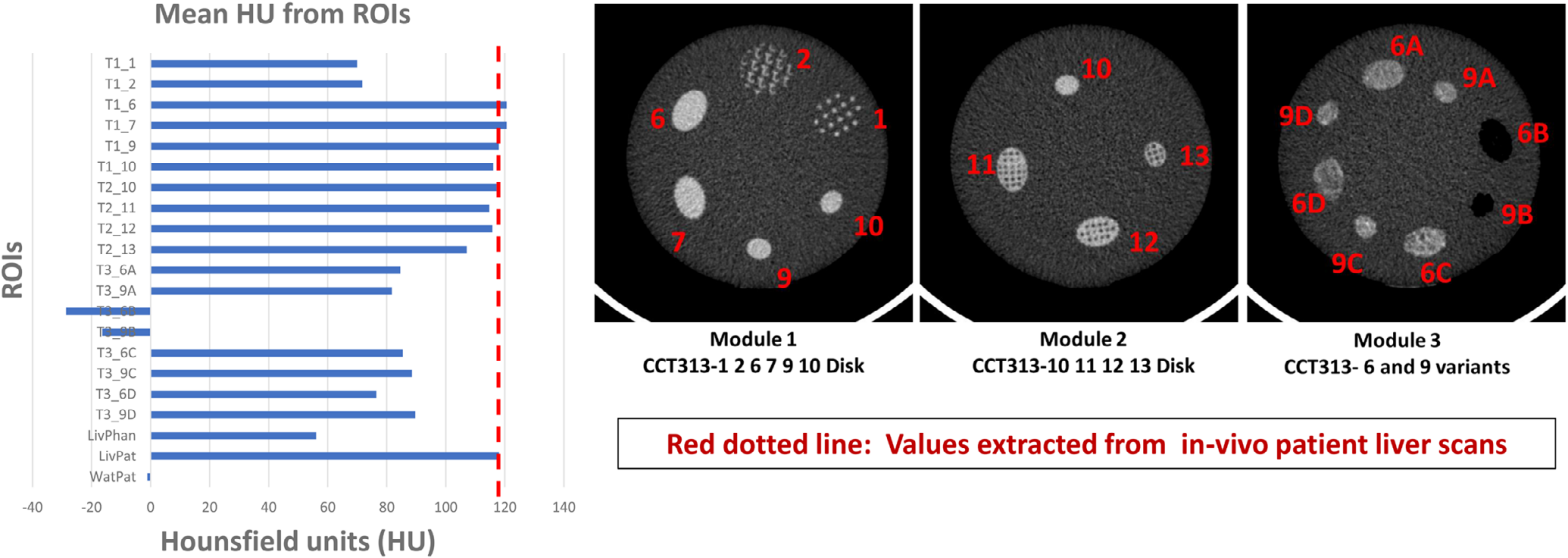
Material equivalence of the radiomics phantom material to the in‐vivo liver scan.

The densities of individual ROI in the phantom ranged from −30 to 120 HU compared to the range of 0–118 HU in a patient liver. Except T3_6B and T3_9B, the HU from each individual ROI in phantom was close to the average of HU among four ROI in a patient liver. This implies that the phantom material used for the construction of the phantom is representative of biological CT tissue densities (here liver tissue) (Figure 4).

### 3.2 Texture equivalence of the radiomics phantom to in‐vivo liver tissue

**FIGURE 5:**
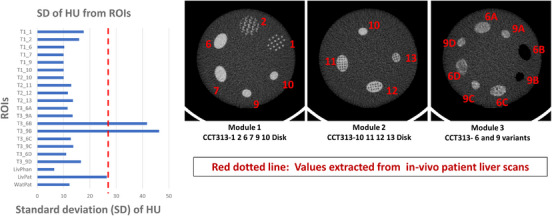
Comparative assessment of material and pattern design in capturing the in‐vivo liver scan setting. Excluding T3_6B and T3_9B, the standard deviation of mean values was larger in the patient liver scan than in the individual ROI of the phantom.

Except for T3‐6B and T3‐9B, disks of module 3 which represent changes in material composition, that is, the patterns are made up of interspersed 3 mm diameter polypropylene spheres, the standard deviation (SD) from each individual ROI in phantom was smaller than the average of HU SD among four ROI in a patient liver. (Figure 5). Considering the technical difficulties in developing the inserts of module 3, this observation implies that materials used in modules 1 and 2 should be further considered to create textures which are equivalent to the textures extracted from the patient liver scan. Patterns of module 1 and 2 are also more readily reproducible in future constructions of the phantom.

### 3.3 Identifying texture patterns that have texture metrics comparable to water phantom, liver phantom, and in‐vivo liver scan

**FIGURE 6:**
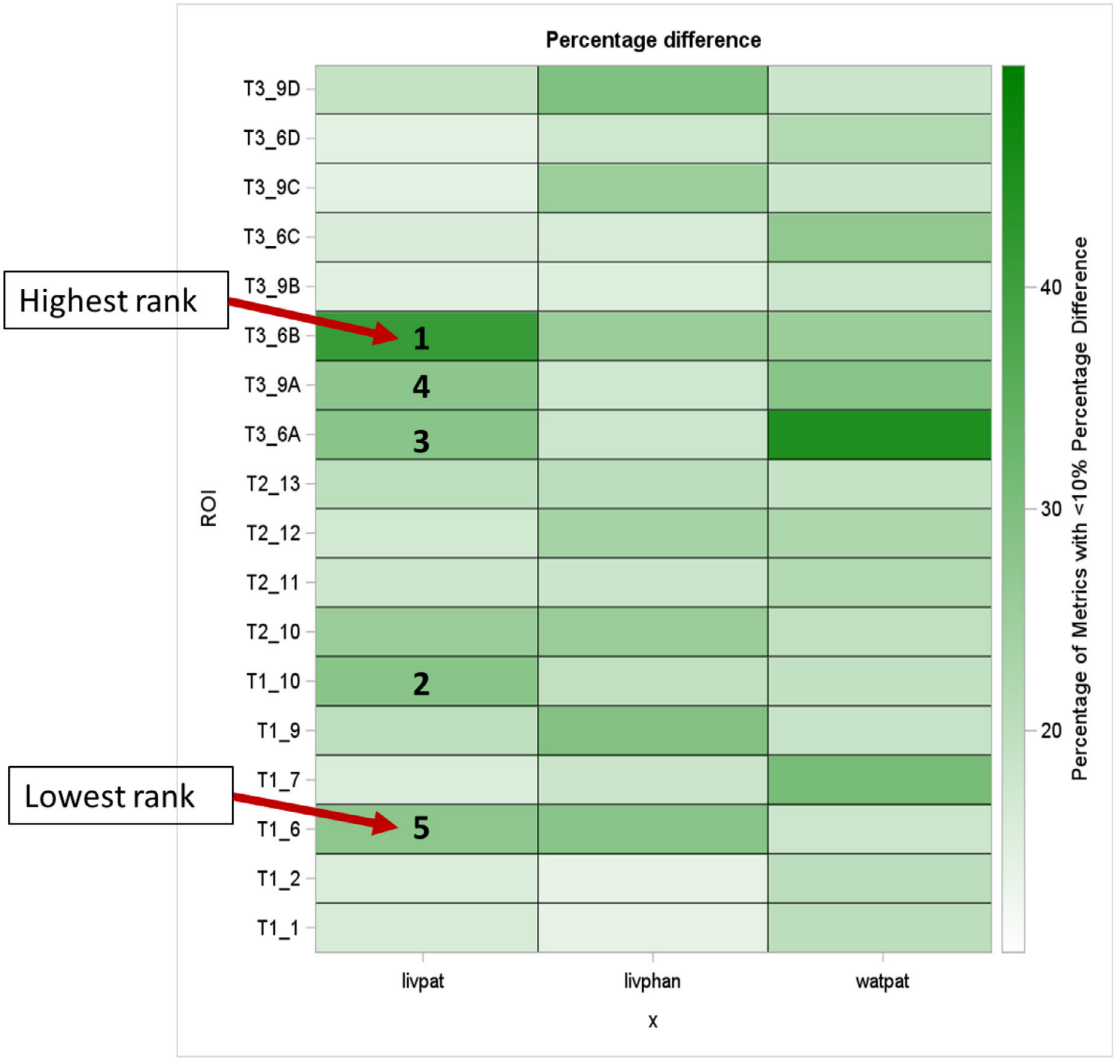
Heatmap of percentage of radiomic metrics with less than 10% change in difference compared to the standards, that is, liver phantom, in‐vivo liver scan and water phantom across the 18 ROI.

**FIGURE 7:**
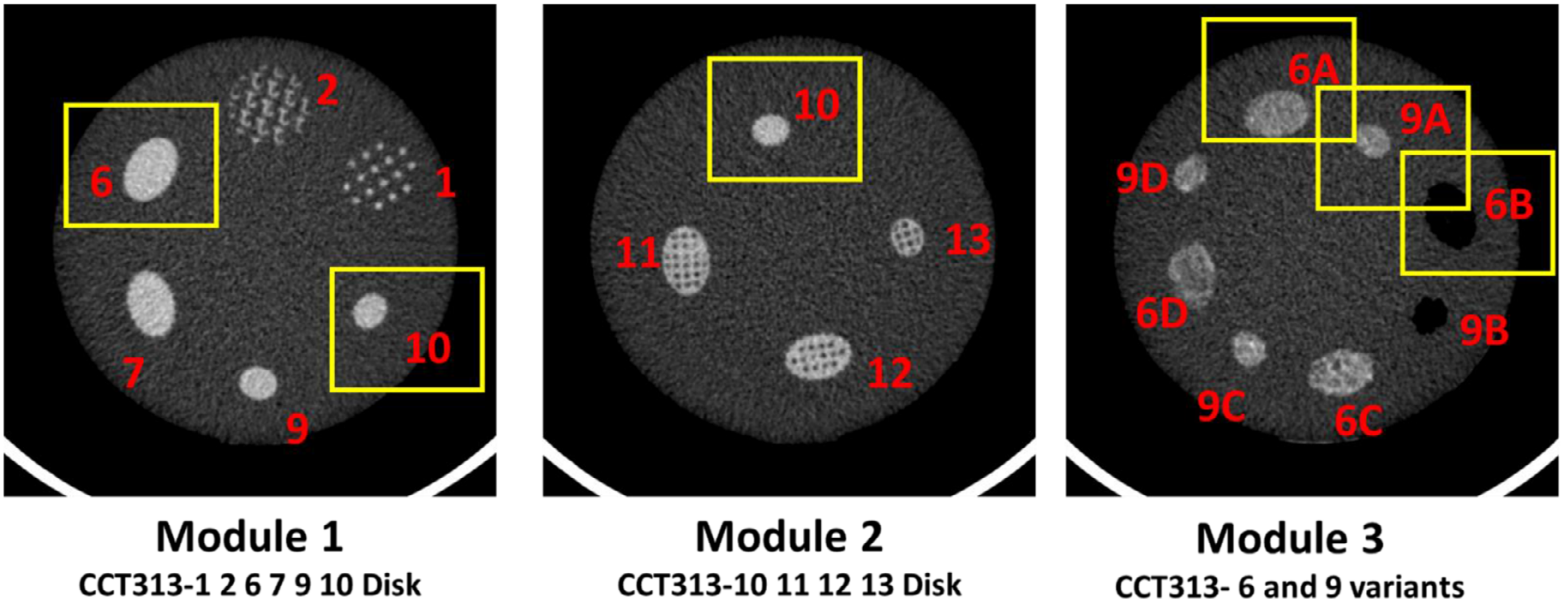
Shortlisted texture modules (highlighted in a yellow box) with maximum number of radiomic metrics with less than 10% change in difference compared to the standards, that is, liver phantom, in‐vivo liver scan, and water phantom across the 18 ROI. The radiomics phantom (The Phantom Laboratory, Salem, NY), consisted of three modules, namely, module CCT313 with patterned disks labelled by the manufacturer as 1 2 6 7 9 10 (Module 1), module CCT313 with patterned disks 10, 11, 12, 13 (Module 2) and module CCT313‐ with patterned disks that are variants of 6 and 9 (Module 3).

The heatmap of the percentage change analysis of the various patterns featured within modules 1, 2, and 3 with respect to the standards, that is, water phantom, liver phantom, and in‐vivo liver scan, revealed texture patterns that showed closest similarity (Figure 6). Each unit in the heatmap shows the percentage of metrics with less than 10% percentage difference among ROI and the standard. For example, Patterns 6 and 10 from Module 1, Pattern 10 from Module 2 and Patterns 6A, 9A, and 6B from Module 3 showed very high similarity across majority of the radiomics metrics observed in the ROI from the in‐vivo liver scan. Despite the mean HU of Pattern 6B being drastically different from that of in‐vivo liver mean HU, ∼50% of the radiomic metrics in pattern 6B from Module 3 showed less than 10% change compared to the same radiomic metrics extracted from the ROI for the in‐vivo liver scan (Figure 7).

### 3.4 Texture patterns that assess variation in higher order texture metrics

**FIGURE 8:**
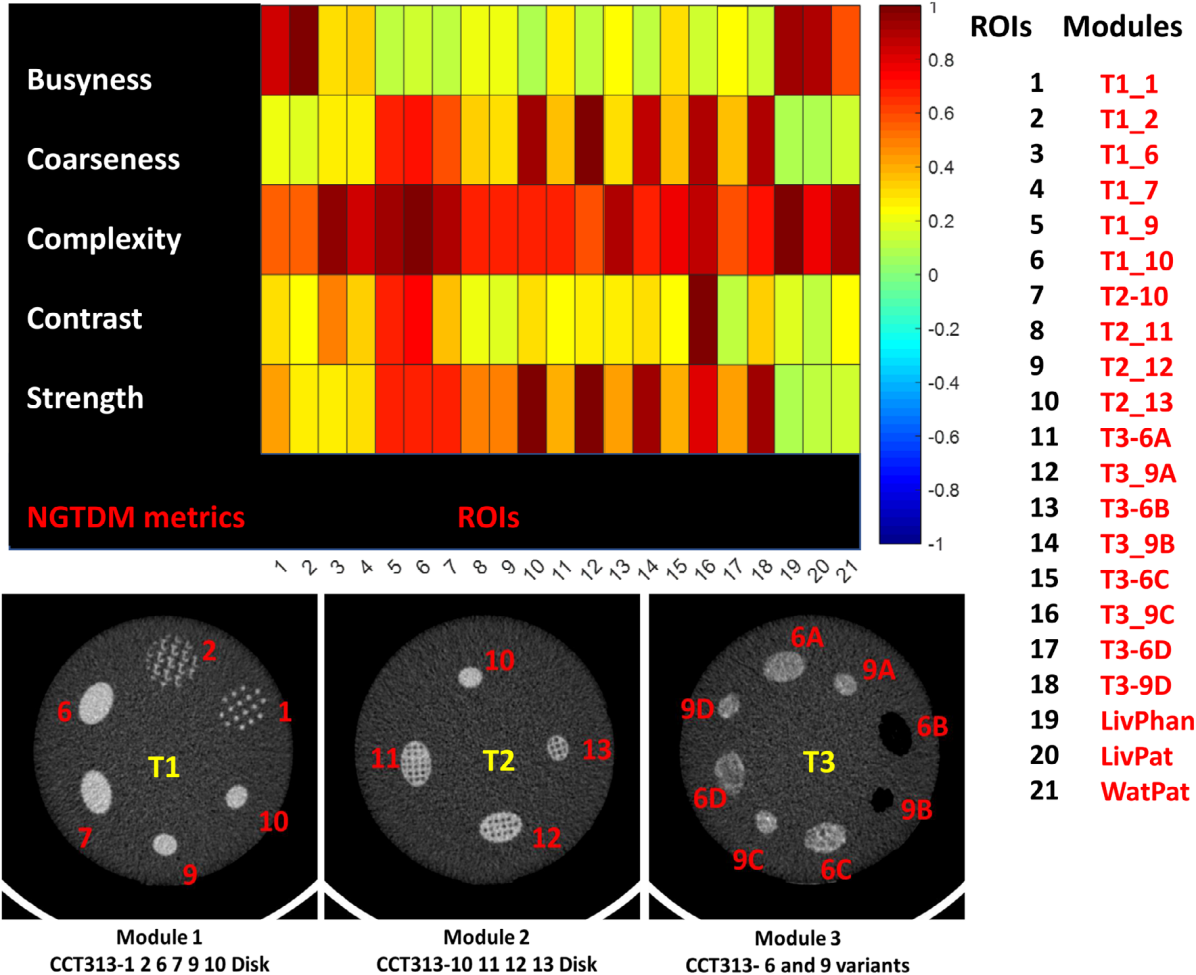
The heatmap of the five NGTDM feature values showing strong dependence on the texture pattern design. The range of measured values in the phantom VOI covered the values seen in the patient liver.

The heatmap of the five NGTDM feature values depended strongly on the insert design. The measured values in some of the phantom ROI (e.g., Patterns T1_1 and T1_2 from Module1) were found to be very similar to the values seen in the patient liver, as opposed to the water phantom, where the HU values are considerably different compared to liver (patient liver ROI comprised of only liver parenchyma with no inclusion of blood vessels or biliary systems). This implies that the pattern design is relevant for assessing second order radiomic metrics such as NGTDM, within the clinically observed range of variation for these values (Figure 8).

## 4 DISCUSSION

The reproducibility and comparability of radiomics features is still a matter of debate and additional standardization efforts are warranted.^26^ Standardized imaging objects such as phantom with the capability of comprehensively and accurately evaluating imaging and analysis procedures are needed for facilitating quality assurance standards and definitions of benchmark parameters for pathology‐specific imaging applications. Prior studies have reported on phantoms that capture the spatial heterogeneity within anatomical regions of human physiology. However, most of the existing phantoms are limited in their abilities to repeat and reproduce the heterogeneous nature of the tumor particularly in multicenter studies. Recently, Gallivanone et al., reported the reliability of radiomics metrics using a novel multimodality image phantom suitable for PET, CT and multiparametric MRI imaging.^27^, ^28^ However, due to the limitations of the process of creating the phantom, 3D digital images of the synthetic lesion were not obtained. While the use of a multimodality phantom is valuable for cross‐imaging radiomics validation, results from our study, could help identify 3D printed texture patterns that could be used in such phantoms. Future studies adopting such phantoms might support a more comprehensive validation of radiomic metrics, particularly higher‐order statistical measures of texture.

Literature reports that a combination of liquids, gels, and solids are typically used for creating heterogeneous phantoms and that these “standardization objects” could be critical to ensuring stable radiomics models across institutions.^29^ Our data suggests that the clear urethane material used for the construction of the phantom is representative of CT tissue densities of liver tissue. With the evolution of advanced image analysis such as radiomics, standardization efforts to ensure the best possible repeatability and reproducibility of the extracted features will require advanced phantoms with reliably detailed features, particularly texture. In module 3 of the phantom, we explore the feasibility of using additional materials such as acetal shavings, polypropylene spheres and insulated glass to create texture patterns within the clear urethane. While Pattern 6B from Module 3 portrays radiomic metrics with the highest similarity to in‐vivo liver scans, the process of creating Pattern 6B is complex and subject to poor repeatability in construction. Although Pattern 6B is similar in shape and size with the Pattern 6 from Module 1 the volume is interspersed with 3 mm diameter polypropylene spheres, making it difficult to recreate the exact consistency in a future phantom. However, a close approximation is possible, and the effect of that variation on the actual radiomics values is not known. In comparison, Patterns 6 and 10 from Module 1, Pattern 10 from Module 2 which also showed comparable similarity to in‐vivo liver scans, are comparatively easy to construct and reproduce in a future phantom. On the other hand, Pattern 6A from Module 3 portrays radiomic metrics with the highest similarity to water phantom scans. Similar in shape and size to Pattern 6B, volume of the Pattern 6A from Module 3 was interspersed with .010′ acetal shavings. Again, as in the case of Pattern 6B, it is difficult to recreate the exact consistency in a future phantom. However, in the current version, approximately 40% of radiomic metrics show less than 10% difference in their values between Pattern 6Aand the water phantom. In comparison, Pattern 7 from Module 1 showed comparable similarity in texture values to the water phantom scans. In both scenarios, that is, comparing Pattern 6B from Module 3 to the in‐vivo liver scans or comparing Pattern 6A from Module 3 to the water phantom scans, although the difference in average HU values were large, the difference in higher order texture metric values were small. This observation further supports the value of exploring of texture patterns suitable for designing a radiomics phantom, even though the mean HU is not suitable for the organ.

The NGTDM quantifies the difference between a gray value and the mean value of its neighborhood within a set distance. These metrics are usually difficult to evaluate using conventional phantoms. Our findings showed strong dependence between the insert design and five NGTDM feature values, namely, busyness, coarseness, complexity, contrast, and strength. In addition, the range of measured values in the phantom ROI covered the values seen in the patient liver. This implies that the pattern design is relevant for assessing second order radiomic metrics such as NGTDM, within the clinically observed range of variation for these values (Figure 8).

There are several limitations to this study. First, while our phantom was designed with multiple components and compared with patient data. The scope was limited to liver tissue. Inserts simulating other types of tissue should be added in future studies. Even from a feature extraction standpoint, we chose to analyze simple cylindrical ROI which may not be representative of the varying morphology of clinical tumors. This was done to focus on evaluation of texture features. In future studies using the same phantom, a thorough radiomics reliability study using multiple vendors, imaging settings and features (size, shape and texture) is warranted. The ability to capture more sophisticated shapes may improve the usefulness of the phantom design. From a construction standpoint, the contrast texture created as a result of the difference in the acrylic based 3D printed materials and the clear urethane provided reasonable performance. The capture of low‐contrast textural variations was weak. In future studies, more advanced 3D‐printing technologies using multiple materials to capture low‐contrast textural variations, as seen in in‐vivo will be explored. Such technologies will aim at making reliable patterns, particularly as used in Module 3, that can be used in future multi‐institution radiomics studies. Regarding texture analysis, to eliminate the variations from the HU range of different CT scanners, a fixed range of HU [−1000, 1500] with a fixed number of bins, that is, 10, as suggested by IBSI was used. However, this could be a source of variation and its impact on the variation is still being explored. Therefore, although beyond the scope of this paper, tissue‐specific, disease‐specific thresholds and quality assurance processes accounting for these biases are crucial prior to the clinical translation of radiomics. Therefore, a reliability assessment of the radiomic metrics using the developed phantom is warranted to systematically evaluate the robustness of radiomic metrics under different imaging conditions that have been reported to affect radiomics results including but not limited to varying slice thickness, dose, tube voltage and reconstruction algorithm. For our analysis, in identifying texture patterns that have texture metrics comparable to water phantom, liver phantom and in‐vivo liver scan, we choose to account for metrics with less than 10% difference among ROI and the standard. Our choice of the 10% cutoff was based on prior published work in radiomics harmonization research.^30^ Currently there is no standard cutoff established for this comparison. Lastly, the in‐vivo comparisons were made using ROI from only a single patient's liver scan because our clinical collaborators had the IRB to scan only one patient for this study. In future studies, additional liver scans should be considered to account for a wider heterogeneity of liver tissue properties. In the study, to reduce the drastic variability in tissue properties, care was taken to avoid regions with obvious blood vessels and biliary systems, when the liver parenchyma ROI were segmented.

To summarize, published literature reports poor generalizability of radiomics studies, particularly those conducted with multicenter settings, possibly due to the confounding effect of the variations in image acquisition, reconstruction, and processing parameters across the different institutions. This observation warrants the need for assessment of radiomics reliability. Phantoms have been used as standardization objects to reduce the effect of confounding variables; however, the currently available radiomics phantom design is preliminary and lacking in its scope to reliably evaluate radiomics for clinic translation. Therefore, despite the limitations, we have built a preliminary radiomics phantom using materials covering a wide span of textures seen in liver CT images. We built textural patterns using diverse methods to capture the radiological heterogeneity in gray‐levels (texture) seen within clinical liver scans. This is an impactful contribution to the CT radiomics knowledge and community to increase its reliability in multicenter analysis. Our results suggested that various radiomics features have material and pattern dependent effects and therefore, it is important to select appropriate materials and patterns that can provide close range of radiomics features to the observation in‐vivo. In addition, in the long run, the designed phantom presents a preliminary opportunity for investigating reproducibility of radiomics features, using multiple vendors, imaging protocols and tissue type to aid in the reliable assessment of radiomics. Maturation of the phantom design and construction detailed in this study may provide a preliminary guide to better understanding the fundamentals of constructing a radiomics phantom, which may improve the generalization of findings in the rapidly growing field of radiomics.

## AUTHOR CONTRIBUTIONS

Bino Varghese— primary author of the paper, idea development, data processing, and analysis; Steven Cen— statistical analysis; Kristin Jensen— imaging protocol development and acquisition of phantom scans; Joshua Levy— idea and phantom development; Hilde Andersen— imaging protocol development; Anselm Schulz— guidance on clinical radiation physics and providing patient scans; Xiaomeng Lei— organized and curated patient data for statistical analysis; Vinay Duddalwar— guidance in the area of radiomics; David Goodenough— overall guidance and provided the clinical insight and context to the study. All the authors reviewed the manuscript and approved the final submission.

## CONFLICT OF INTEREST STATEMENT

David Goodenough is a Consultant to The Phantom Laboratory, Salem, NY. Joshua Levy is an employee and Founder of The Phantom Laboratory, Salem, NY.

## ETHICS STATEMENT

For all procedures performed in studies involving human participants written informed consent was obtained and has secured all the required IRB approvals.
